# Parental involvement in robot-mediated intervention: a systematic review

**DOI:** 10.3389/fpsyg.2024.1355901

**Published:** 2024-07-09

**Authors:** Adriana Piccolo, Carmela De Domenico, Marcella Di Cara, Carmela Settimo, Francesco Corallo, Simona Leonardi, Caterina Impallomeni, Emanuela Tripodi, Angelo Quartarone, Francesca Cucinotta

**Affiliations:** IRCCS Centro Neurolesi Bonino Pulejo, Messina, Italy

**Keywords:** robotics, parenting, caregivers, children, pediatric rehabilitation

## Abstract

**Introduction:**

Over the years, the conceptual approach to pediatric rehabilitation has reevaluated the parent's role in the therapeutic process, considering parental involvement as a necessary condition for the effectiveness of the intervention. In the field of pediatric intervention, the therapeutic use of robots represents a growing clinical interest, but the feasibility and applicability of these robotic interventions, including those involving parents, remain unclear. This systematic review aims to investigate parental involvement in robot-mediated interventions (RMI) for children and adolescents in the current literature. Our main goal is to analyze and summarize all existing studies to discuss and draw future research directions and implications for clinical practice.

**Method:**

After collecting results from 1,106 studies, the studies selected were analyzed using thematic analysis. The literature review was conducted in accordance with the PRISMA guidelines by searching databases such as PubMed and Web of Science until 07 February 2023. Studies that met the following inclusion criteria were included: (1) the use of a robot as a therapeutic-rehabilitation tool and (2) the presence/involvement of parents/caregivers in child-robot therapeutic sessions.

**Results:**

A total of 10 articles were included. The extracted data included study design, participant characteristics, type of robot used, outcome measures, aim, and type of intervention. The results reveal that parental involvement in RMI could be feasible and useful in improving intervention efficacy, particularly in improving the child's social-communicative abilities and the caregiver's educational skills.

**Discussion:**

RMI intervention with parental participation could be a useful therapeutic strategy in pediatrics. However, to date, few studies have investigated this specific topic, and the reported results may enhance future research to understand its effectiveness in specific areas of use.

**Systematic review registration:**

identifier: CRD42024553214.

## 1 Introduction

The recent debate and a broad body of scientific literature support the idea that parenting has a very important role in a child's mental health. From the first few months of life, a large body of evidence suggests that early, secure attachments with parents lay the foundation for secure attachments in adulthood (Murray, 1990; Stein et al., 1991). Positive, proactive parenting is associated with high academic competence and good self-esteem and can be protective against later challenging behavior (Gardner et al., 2007) and substance misuse (Baumrind, 1985; Hawkins et al., 1997). Moreover, the caregiver environment during infancy and parenting interaction variables have been shown to explain up to 30%−40% of externalizing disorders and antisocial behavior in a child at school entry (Patterson et al., 1989; Shaw et al., 2001). In addition to the prevention of mental disorders across the lifespan (Fonagy, 1998), accumulating evidence would seem to suggest that parental involvement in pediatric treatment programs may influence the outcome, increasing parenting self-efficacy and reducing the perceived stress levels of caregivers (Gatta et al., 2016). It is thus possible to increase learning opportunities in all contexts, training the parent with valid educational strategies (Wainer et al., 2017). For all these reasons, more and more investments are being made in parent-mediated intervention programs to allow the opportunity to extend the child's learning to daily life contexts, increasing the consistency of management strategies. Besides, studies examining parent-mediated interventions vary by program, population, location, and mode of delivery. To date, numerous studies have investigated the effectiveness of specific interventions that involve, in various ways, the presence of the parent. Furthermore, the principal evidence-based treatment in this field is the parent training program, which consists of structured behavioral interventions to improve children's behavioral and emotional management (Benedetto, 2005). However, the term “*parent training”* can be used to refer to a heterogeneous set of therapeutic interventions, such as, group programs for the management of challenging behaviors (Postorino et al., 2017), video-modeling sessions for communication skills enhancement (English et al., 2017), and psychoeducation (Steiner et al., 2012), in the absence of an unambiguous and shared definition.

The more diffuse programs in clinical practice, known as parent training (PT), focus on improving the parent–child relationship, using the parent as an agent of change for the child's behavioral problems, and effectively reducing disruptive behavior (Forehand et al., 2013). PT therapists aim to increase positive parent–child interactions by teaching specific skills, such as emotional communication and coherent discipline strategies (Kaminski et al., 2008). This kind of intervention is founded on the theoretical assumption that negative behaviors are learned and sustained by the positive and negative reinforcements children receive from their parents and their surrounding environment (Serketich and Dumas, 1996). Studies have shown that this intervention has successfully modified children's behaviors, decreasing verbal aggression and self-destructive and oppositional behaviors (Rose, 1974; Wade et al., 2008) and bolstering emotion regulation (Sukhodolsky et al., 2016), with greater generalizability and longer retention of treatment benefits (Blizzard et al., 2018). PT has proven effective in different neurodevelopmental disorders, including attention-deficit/hyperactivity disorder (Chronis et al., 2004; Webster-Stratton et al., 2011; Zwi et al., 2011), intellectual disability (Coren et al., 2018), autism spectrum disorder (Wetherby et al., 2014; Bearss et al., 2015; Roberts et al., 2016), and language impairment (Roberts et al., 2019).

In recent years, developments in robotic technology have attracted the attention of the educational and rehabilitative sectors. Robot intervention has proven effective in helping children with developmental disabilities, in particular children with autism spectrum disorder (ASD), where the robot may be able to elicit more active communication and engagement compared to an unfamiliar human speaker (Wood et al., 2013). In fact, this emerging research field highlights the social aspects of human–robot interaction and is being increasingly used as a complementary therapy to improve social communication skills in children with ASD (Thill et al., 2012). Most exploratory studies have focused on the participants' responses toward the robot during interaction (Pennisi et al., 2016), on their potential to improve learning (Akalin and Loutfi, 2021), or to teach or make the child perform a specific skill (Diehl et al., 2014; Castillo et al., 2018).

The enthusiasm for robot-mediated intervention (RMI) is probably because they can act as particularly motivating mediators and assistants in developmental rehabilitation (Wainer et al., 2010; Lee and Obinata, 2013). All these findings supported the use of social robots in pediatric interventions, and the several ways of interacting with robots made it possible to employ them for multiple purposes. However, this has overlooked the possibility of analyzing the usefulness of this tool in improving and sustaining parental support or parental implementation interventions, underestimating its rehabilitative role. In this framework, parents are not only key informants for therapists and researchers but also an important resource in the therapeutic setting, capable of producing overall effects on the child's development (Green et al., 2010). Indeed, it seems essential to remember how parents may influence a wide range of interventions on children's social communication (Laski et al., 1988), sleeping habits (Reed et al., 2009), joint attention (Kasari et al., 2010), and adaptive behaviors (Kroeger and Sorensen, 2010).

Therefore, based on the assertion that parent-focused interventions promote greater generalizability of learned skills and longer maintenance of treatment benefits (Steiner et al., 2012), this systematic review aims to analyze parental involvement in RMI for children and adolescents in the current literature. Our main goal is to analyze and summarize all existing studies to discuss and draw future research directions and implications for clinical practice.

## 2 Materials and methods

### 2.1 Data source and search strategy

**Figure 1:**
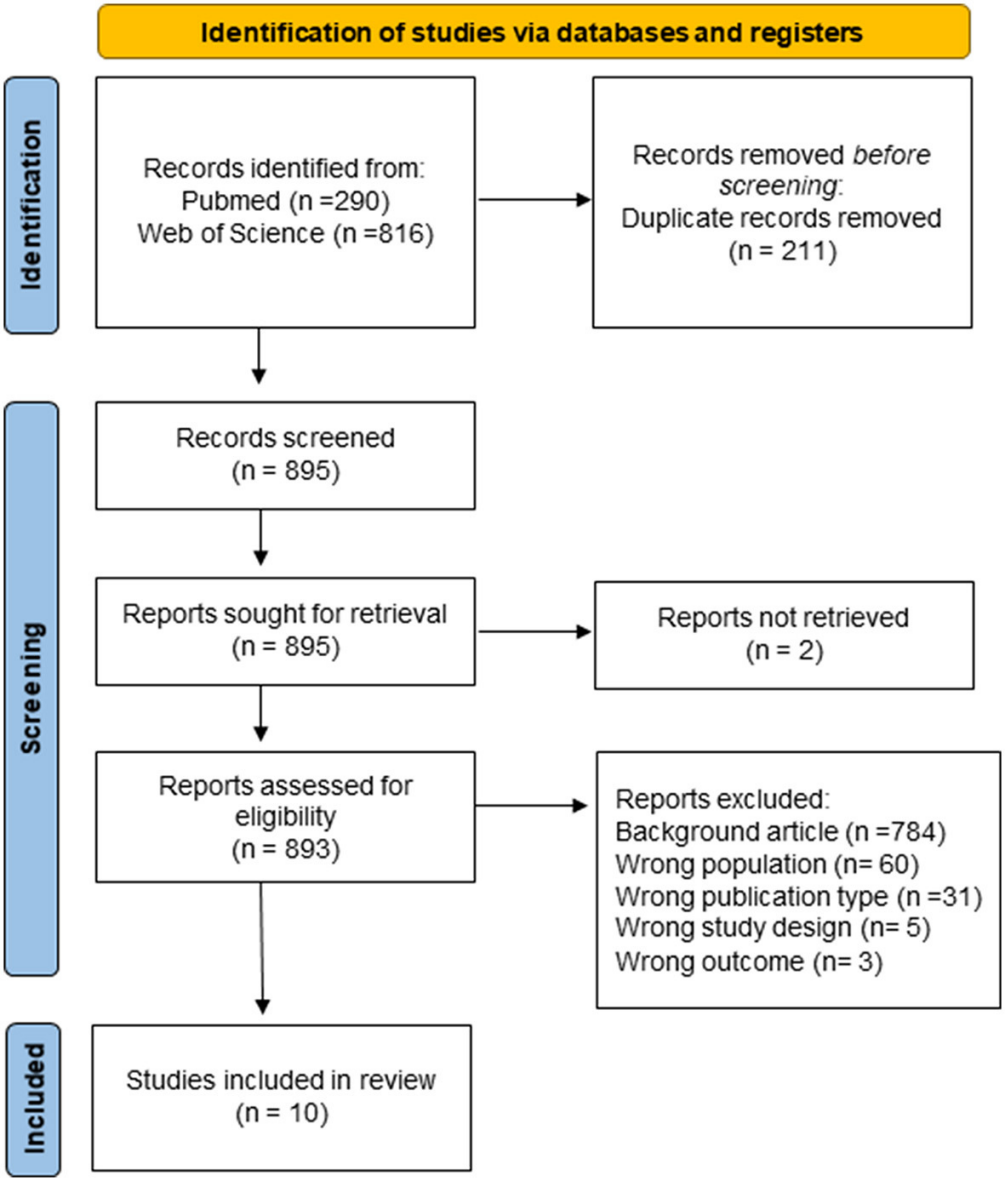
Preferred Reporting Items for Systematic Reviews and Meta-Analyses (PRISMA) flow diagram for study selection.

Literature reviews were conducted in accordance with PRISMA guidelines (Tricco et al., 2018) by searching PubMed and the Web of Science until 07 February 2023 (see Figure 1). To expand results, we combined two main categories of keywords (ROBOT and PARENTS) with the use of various synonyms in the search query or the respective MeSH terms. We also manually searched the references to the included articles or related reviews and extracted any additional relevant titles. The extracted data were blindly analyzed by two authors (AP and MDC) independently. Whenever ratings were discordant, a third investigator (FC) analyzed the result and then reached a consensus.

### 2.2 Study selection

The literature search yielded a total of 1,106 results. After removing 211 duplicates, we had 895 results for screening. Studies were included if they met the following inclusion criteria: (1) robot as a rehabilitation-therapeutic tool; (2) presence/involvement of the parents/caregiver within child–robot therapeutic sessions. All titles and abstracts were initially screened to exclude studies that did not meet even one inclusion criterion. Articles in all languages were accepted and later excluded if a translated article could not be achieved.

After the screening, the full-text review exclusion criteria for the abstract and title review were reapplied, and the following exclusion criteria were taken into account: (1) sample age >18 years old and (2) commentaries, case reports, and reviews. All articles were evaluated by title, abstract, full text, and specificity of the topic. Two articles were excluded from the 895 articles included for screening because reports were not retrieved. There were 893 reports evaluated for eligibility. Of these, 784 articles were excluded for incorrect background, 60 for incorrect population sample, 31 for incorrect publication type, 5 for wrong study design, and three for wrong outcome. The full-text review yielded a total of 10 included articles. Extracted data included study design, participant's characteristics, type of robots used, focus and type of intervention, and psychological outcomes.

## 3 Results

A total of 10 studies presented parental involvement in RMI. See Table 1 for more details. These studies used several parent-mediated interventions, and an active parental role was not always planned. These studies were published between 2020 and 2023. Four studies were conducted in Europe (id #1-#2-#8-#10), three in Asia (id #3-#4-#7), two in the United States (id #5 #9), and one in Australia (id #6). The location of the sessions varied among the studies: six studies provided specific training and took place mainly in rehabilitation centers; two studies were conducted in tertiary pediatric facilities, and two were conducted in home settings. Sample characteristics and range of age differ significantly among these studies; they included between 5 and 25 children, ranging in age from 3 to 16 years. Only three studies were randomized controlled trials (RCTs), while the remaining were within-subject or qualitative. The sample from the included studies included the following disorders: autism spectrum disorder (n.5), visual impairment (n.1), and other medical conditions in pediatric patients (n.2). Furthermore, two studies focused on children with typical development. We have summarized all sample characteristics and study design in Table 2, and the aims, outcome measures, and main results in Table 3. To better assess and summarize all the results, we analyzed all the studies according to population diagnostic features.

**Table 1:** The articles selected and numbered.

**ID**	**Author**	**Year**	**Title**	**Journal**
#01	Van den Berk-Smeekens et al.	2020	Adherence and acceptability of a robot-assisted Pivotal Response Treatment protocol for children with autism spectrum disorder	*Scientific reports*
#02	Rocha et al.	2021	Accembly at Home: Accessible Spatial Programming for Children with Visual Impairments and their Families	*Idc '21: proceedings of interaction design and children 2021*
#03	Amirova et al.	2023	Effects of Parental Involvement in Robot-Assisted Autism Therapy	*Child | humans*
#04	Rakhymbayeva et al.	2021	A Long-Term Engagement with a Social Robot for Autism Therapy	*Frontiers in robotics* *and AI*
#05	Scasellati et al.	2018	Improving social skills in children with ASD using a long-term, in-home social robot	*Science robotics*
#06	Butchart	2021	Child and parent perceptions of acceptability and therapeutic value of a socially assistive robot used during pediatric rehabilitation	*Disability and rehabilitation*
#07	Gvirsman et al.	2020	Patricc: A Platform for Triadic Interaction with Changeable Characters	*Social robots | children*
#08	Tolksdorf et al.	2021	Comparing the Effects of a Different Social Partner (Social Robot vs. Human) on Children's Social Referencing in Interaction	*Frontiers in education*
#09	Okita et al.	2013	Self-Other's Perspective Taking: The Use of Therapeutic Robot Companions as Social Agents for Reducing Pain and Anxiety in Pediatric Patients	*Cyberpsychology behavior and social networking*
#10	Otterdijk et al.	2020	The effects of long-term child–robot interaction on the attention and the engagement of children with autism.	*Robotics*

**Table 2:** Sample characteristics and the study design of selected studies.

**ID**	**Diagnostic features**	**Sample size: n. children; n. parents**	**M: F**	**Children years range (M; SD)**	**Study design**	**Robot**
#1	ASD	25 children; NS parents	4:1	3–8 (6.24 ± 1.3)	No control group, quantitative/qualitative analysis	NAO Robot
#2	Visual impairments	Seven children; NS parents	4:3	7–14 (9.14 ± 2.3)	No control group, quantitative/qualitative analysis	ACCembly and Dash robot
#3	ASD	16 children; NS parents	7:1	5–12 (6.75 ± 2.1)	Within-subject and quantitative/qualitative analysis	NAO Robot
#4	ASD	11 children; NS parents	11:1	4–11 (6.1 ±2.7)	No control group, quantitative/qualitative analysis	NAO Robot
#5	ASD	12 children; 12 parents	1:0.857	6–12 (9.02 ±1.4)	Pilot study	Jibo robot
#6	Other medical condition	Five children; Five parents	3:2	6–12 (NS)	No control group, quantitative/qualitative analysis	NAO Robot
#7	TD	18 children; 18 parents	1:1 8:1	1–4 (NS)	No control group, quantitative/qualitative analysis	Patricc platform
#8	TD	20 children; NS parents	7:3	4–5 (5 ± 0.6)	RCT	NAO Robot
#9	Other medical condition	18 children; 18 parents	0:1 0:1	6–15	RCT	Paro Robot
#10	ASD	Six children; NS parents	3:1	3–8 (5.17 ± 1.5)	RCT	NAO Robot

ASD, autism spectrum disorders; TD, Typical Development; NS, not specified; M, Male; F, Female; RCT, randomized controlled trial.

**Table 3:** Aims, outcome measures, and main results of the selected studies.

**ID**	**Study protocol and task type**	**Aims**	**Outcomes measures**	**Results**
#1	A robot-assisted pivotal response treatment (PRT) protocol is composed of 20 sessions weekly: 14 parent-child sessions, four parent sessions, and two child's teacher sessions. The sessions with the robot lasted 15–20 minutes.	a) CHILD affect and likability of the robot and b) Parent ratings of robot-assisted sessions	a) Children raters: Child 5-point Visual Analog Scale (VAS); b) Parents raters: Session Rating Scale (SRS) (Duncan et al., 2003); and 100-point VAS on ability robot communication; and robot as an additional value.	a) Child affect and likability of the robot were positive; the high-severity ASD group showed lower affect scores; the preschool-aged group had lower mean robot likability scores compared with the school-aged group. b) The parent ratings of the robot's acceptability were mainly positive.
#2	The researcher delivered the coding kit to families. Children and parents interacted with the session, starting by following the guidebook and freely exploring the setup.	a) Children's computational thinking learning, b) Engagement, and c) Parent-child interactions and mutual engagement to support qualitative play.	Video-coding analysis and parent interviews.	a) CHILDREN leveraged fundamental computational thinking concepts to solve spatial programming challenges. b) Most children engage with assembly in a structured and goal-driven way by following the activities in the guidebook. c) Parents took on different roles as mediators, some actively teaching and scaffolding, others learning together with their child.
#3	7–10 of 15-min sessions with the NAO robot plus, as usual, treatment that includes traditional methods of autism therapy (art, music therapy, and others) for a 21-day period. Parents were invited to attend the sessions, but it was not mandatory for them to be present.	Socio-behavioral engagement in parental involvement for RMI	a) 11 measures were analyzed through coded videos (Kim et al., 2012; Rudovic et al., 2017); some of these involved the use of a Likert scale, while others collected data on the frequency and duration of a target behavior: engagement, valence, engagement time, eye gaze time, affection, curiosity, aggression, chest button, stereotyped behaviors, smiles, and words. b) parents' semi-structured interviews.	a) There were no significant differences found between sessions with or without parents b) most parents observed positive changes in their children's verbal and non-verbal behaviors; the most reported was maintaining eye contact with the robot.
#4	The intervention consisted of 30-minute sessions per day for a month, during which the triadic interaction between the child, the parent, and the robot was evaluated. The robot modeled the interaction through feedback in six interactive games played on a display.	a) Increasing engagement and valence scores with the robot over multiple sessions. b) Using activities that are familiar to each child will lead to increased overall engagement from session to session.	a, b) 8 measures analyzed through coded videos (Kim et al., 2012; Rudovic et al., 2017): engagement, valence, engagement time, eye gaze time, and their respective mean value. Parents had semi-structured interviews.	a) No significant differences were found between each session. The parents seemed satisfied with the therapy itself. Their suggestions include equipping NAOs with more active and personalized behaviors. b) Mean eye gaze duration, mean engagement, and engagement duration resulted significantly higher in familiar sessions.
#5	Up to three sessions with the robot with an average session length of 33 min.	a) Investigate how a social robot could provide behavioral intervention for children with ASD outside of clinical contexts. b) Evaluate whether this intervention could provide improvements in the social-communicative skills of children with ASD.	The child's joint attention was measured using the naturalistic model of Bean and Eigsti (2012) and included, in 4-time points, an evaluation of the interaction during the game outside the robotic session. Within the sessions with the robot, attention was measured using an attention-tracking subsystem. The software recorded the poses and orientations of the head using an RGB camera and face-tracking algorithms. The parental assessments were conducted through interviews with responses on a 5-point Likert scale and were proposed at the end of each intervention session.	a) An improvement in joint attention was found in the test phase but not in the comparison between pre-test and post-test. b) Parental interviews reported an increase in the child's social skills on the last day of the intervention compared to the first day, both in interaction with the parent and with other people.
#6	Three 8-minute learning sessions: in two of the sessions, the toddler and parent interacted with Patricc (ROBOT1, ROBOT2), and in one session, they interacted with a tablet displaying an interactive video of the same robot.	Capture the experiences of parents and children according to 5 thematics: a) affective influence, b) independence, c) preference for human interaction, d) accessibility of therapy (only for parents), and e) familiarity with technology.	Semi-structured, in-depth child and parent interviews	a) Participants viewed the affective influence (involvement and motivation) during the rehabilitation activity with a socially assisted robot. b) Parents identified that the robot could increase the child's independence in exercises. c) Both child and parent participants identified the importance of the therapeutic relationship, which the socially assistive robot was not able to replicate. d) Parents identified barriers to accessing therapy, including time and distance from services, but also competing priorities, such as their child's education. e) Parents and children were familiar with technology and did not express difficulty with its use.
#7	Four sessions of 20–35 min, within a 2-week period. The children, accompanied by a parent, were randomly assigned to a parallel learning situation with either (1) the social robot or (2) the human interlocutor.	a) Triadic interaction between the toddler, parent, and robot and b) Qualitative assessments of their experience.	a) Video-coding analysis of the triadic interaction using six gaze measures: the toddler gazed toward the robot, the tablet, or the parent; the parent gazed toward the toddler, the robot, or the tablet b) Parents semi-structured interviews.	a) Patricc gaze data vs tablet were significantly different: increased parent's gaze at the toddler in Patricc's condition; increased mutual gaze at the tablet in the TABLET condition. No difference was found among the changeable characters. b) the robot as a better means of teaching language than the tablet for parents; 50% of parents saw the game with the robot as a parent-toddler activity; most parents affirmed that changing the robot's characters contributed to the activity.
#8	One 30-minute session. The children were randomly assigned to one of two conditions: in the “alone” condition, the patient was engaged with Paro for 30 minutes. In the “together with parent” condition, the patient and parent took turns interacting with Paro for 30 minutes.	a) Children's social referencing in interaction with a social robot and b) Children's social references in a long-term interaction.	All sessions were video recorded, and the infants' gaze behaviors were coded in the direction of their caregivers (Vaish and Striano, 2004).	a) In the human condition, the number of social referrals was significantly lower than when their peers were interacting with the robotic partner. b) Children's social referencing did not decrease in the long term in either condition.
#9	20 sessions of 15–20 minutes, once a week, for a period of 6 months. Participants were randomly assigned to the robot-assisted PRT or treatment as usual. Parents were also involved in the sessions.	a) Pain perceived and b) Emotional anxiety in patients and their parents.	a) Wong-baker FACES Pain Rating Scale (Wong and Baker, 2012) b) Anxiety questionnaire: the State–Trait Anxiety Inventory for Children (STAIC) 21 and State–Trait Anxiety Inventory (STAI), Spielberger (1973)	Patients who engaged with their parents with Paro showed greater reductions in (a) pain and (b) negative emotional anxiety than patients who interacted with Paro alone.
#10	7–10 of 15-min sessions with the NAO robot plus, as usual, treatment that includes traditional methods of autism therapy (art, music therapy, and others) for a 21-day period. Parents were invited to attend the sessions, but it was not mandatory for them to be present.	a) Investigate how a social robot could provide behavioral intervention for children with ASD outside of clinical contexts and b) Evaluate whether this intervention could provide improvements in the social-communicative skills of children with ASD.	All sessions were video recorded and coded by analyzing the direction and valence of the behavior through gaze behaviors and arm/hand behaviors.	Attention and engagement did not decrease over time.

### 3.1 Parent involvement in RMI in autism spectrum disorder

Five publications reported on parent presence in robot-mediated interventions for ASD (ID #1, #3, #4, #5, #10). All the studies' samples included a range of ages between 3 and 12 years; within this range, the most represented age average was 6 years. The population was male dominated. Among those, only one article was a randomized controlled trial; the other reports are single-subject design, within-subject, or qualitative studies. The majority of studies used the Nao robot as a rehabilitation tool for only an average number of ~10 sessions, except for study #10, which includes a follow-up visit. NAO is a humanoid robot equipped with ultrasonic and pressure sensors placed under its feet. It has a multimodal system with four microphones, two speakers, and two video cameras. This system allows vocal synthesis, facial recognition, and spatial localization, allowing the robot to navigate and interact within various environments effectively. Nao robots also speak and ensure a certain degree of non-verbal communication, capturing information about the environment using cameras and microphones. Only one study used the Jipo table robot, equipped with a 360-degree head and body rotation system and software with an overhead camera. This tool can exhibit expressive behaviors through a pair of animated eyes and changing emitted lights. The robot can make eye contact with the participants and follow their points of attention. In addition, another camera recorded the entire intervention session.

The principal aim of all reports was to verify the feasibility and efficacy of RMI as support tools for specific interventions. Specifically, the studies of van den Berk-Smeekens et al. (2020) and van Otterdijk et al. (2020) planned 20 Nao sessions weekly, integrated with Pivotal Response Treatment (PRT); in both studies, the Nao robot was used to reinforce social interaction, self-initiations, and training parents. The first one aimed to verify the feasibility and likability of the robot; the second one, an RCT study, aimed to verify longitudinal attention and engagement in robot-assisted therapy. In addition to those goals, Rakhymbayeva et al. (2021) evaluated the involvement of children with ASD in robotic sessions when comfortable or unseen activities were included in RMI, with the opportunity to share with parents. The children attended 15 sessions, for a maximum of n.10 RMI, which provided a gradual shift toward the most preferred activities in the last session.

Conversely, despite the use of the same robot, only the study of Amirova et al. (2023) evaluated whether and how parental involvement in RMI promoted children's socio-behavioral engagement. They provide 15-min sessions for a maximum of 10 Nao sessions, with and without parents' presence; the activities proposed were predominantly based on applied behavioral analysis, targeted at socioemotional behaviors, with no restricted role for parents. Finally, Scasellati et al. (2018), with the Jipo table robot, aimed to investigate how this social tool could improve a behavioral intervention in a home context and if it could improve social-communicative skills in everyday life. The RMI consisted of interactive games to practice social skills in a home context, organized in 30-min sessions every day for a month with the constant presence of a parent. During the session, Jipo attempts to redirect the children's gaze to the caregiver to improve the triadic interaction and share the gaming experience with the parent.

Video coding of target behaviors, defined according to the outcomes of each study, was the main measure used. Most studies that used video coding have considered the amount of time a child spent looking at the robot and the frequencies of the child's gaze toward the robot (studies #3-#4-#10). Moreover, all three studies attempt to measure the engagement of the child through compliance and affective reactions, including facial expressions and gestures. Other metrics included a visual analog scale (VAS), filled out by the child and parent, and semi-structured interviews. In particular, van den Berk-Smeekens (#1) at the beginning and end of each robot-assisted session used a 5-point VAS, measuring the child's affection for and liking of the robot; the parents completed two VAS lines between 0 and 100 to assess whether the communication of the robot toward the child was clear (robot communication) and whether the robot represented an additional value to the current therapy.

Moreover, Amirova et al. (2023) analyzed the parents' perceptions using semi-structured interviews recorded in audio format. A thematic analysis was used to process the interview data, which allowed for both deductive and inductive themes. Otherwise, in Scasellati (#5), joint attention was tracked during the session using an attention-tracking subsystem. Within the sessions, an RGB camera recorded face and head movement, and software tracked approximated attentional targets according to the poses and orientations of the head. Moreover, to evaluate the child's joint attention, the naturalistic model of Bean and Eigsti (2012) was used. This model, applied four times throughout the study (T_0_-30days before the intervention, T_1_-on the first day of the intervention, T_2_-on the last day of the intervention, and T_3_-30 days after the end of the intervention), includes a play session with the researcher to collect data on gaze following, response to name, greeting opportunity, and recognition of other people's interests. To verify the generalization of behavior, the parents filled out a survey immediately after each intervention session. The survey consisted of an evaluation of responses with a 5-point Likert scale on how the child interacted with the caregiver in the last 24 h and how the child interacted with other people. Finally, they measured the total interactive game score achieved by each patient.

Regarding the results of those studies, despite all studies providing parental participation in RMI, only one study explores the influence of parental involvement. van Otterdijk et al. (2020) (#10) measured the average change of attention and engagement in the activities, both with the robot and the game, and toward other people involved, both parents and therapists, at different stages of treatment (beginning, middle, and end). In this last analysis, they described an increase in attention and engagement toward other humans involved in the sessions, particularly with the parents. In contrast, there was no change over time in the attention given to the therapist alone. The other three studies (#1, #3, and #4) analyzed only the parental observations on the usefulness of RMI for children with ASD. Specifically, van den Berk-Smeekens et al. (2020) (#01) confirmed a highly positive parental evaluation of the NAO robot, with 94.3% indicating that the robot represented an addition to the PRT therapy session.

In the study by Amirova et al. (2023) (#03), the interviews recorded revealed that most parents observed positive and tangible changes in their children's verbal and non-verbal behaviors after the intervention, with the most recurring observation being an increased ability to maintain eye contact with the robot. Specifically, it was observed that children with severe autism touched the button on the robot's chest more often during sessions when their parents were present. This behavior suggests that the presence of parents may provide these children with a greater sense of security, encouraging them to explore the robot more actively. On the contrary, for children with ASD who have verbal communication abilities, the involvement of the parents may not represent an added value, as they may be able to deal with the social situation independently. Finally, the general parent perception considers social robots to be useful tools with therapeutic value.

Rakhymbayeva et al. (2021) (#04) reported no changes in the levels of engagement or valence with the social robot. Additionally, feedback was gathered from five caregivers, who provided therapeutic recommendations based on their experiences. While parents expressed positive attitudes toward RMIs, they also recommended enhancing the interactive quality of the sessions. Specifically, they suggested the use of robots that exhibit more dynamic and active behaviors to foster livelier interactions.

Finally, study #5 (Scasellati et al., 2018) reported an improvement in social skills using an in-home social robot. Specifically, the joint attention performance showed improvement during the experimental intervention. Parental evaluations confirmed significant differences between the first day of intervention and the last one. Parents reported an increase in social skills between children and themselves, including eye contact, more attempts to initiate communication, and more frequent responses to communication bids. The same amelioration in all three behaviors was reported between the child and other people. Regarding engagement, no significant worsening was detected over time. A global ameliorant in all level games was recorded, with a specific gain in the perspective-taking game “*House*,” where all the children reached the most difficult level by the end of the scheduled sessions.

### 3.2 Parent involvement in RMI in visually impaired pediatric patients

Only one study explored an RMI for visually impaired pediatric patients. The study of Rocha et al. (2021; id #2) is a within-subject, qualitative study where n.7 families were enrolled. The child sample was characterized by a range of ages between 7 and 14 years; within this range, the most represented age was 10 years, and the population is male dominated. The parent age range was 34–48 years.

ACCembly was used for intervention: a physical block-based environment that allows children with visual impairments to physically assemble blocks to program spatial actions made visible by a robotic device with multimodal output. The tangible blocks, inspired by those employed by Pires et al. (2020), are equipped with magnets and saliences to favor coupling and are characterized by different colors and pictograms to distinguish the block's actions. Tactile feedback enabled the children to recognize both individual blocks and their sequences. In addition to the tangible blocks, ACCembly comprises a Dash robot, soft tiles to build a map, and an Android application on a mobile device that uses its camera to detect the blocks, interpret their sequence, and send the instructions to the robot. Children and parents interacted with ACCembly, following the guide and freely exploring the configuration. The purpose of this study was to evaluate the learning of computational thinking and parent–child interaction during play. The outcome measures included video-coding analyses of children's and parents' behaviors and interviews with parents. Specifically, they investigated parents' behaviors with a 10-defined role framework proposed by Yu et al. (2020), based on parent interactions throughout the activities.

Globally, all the families evaluated ACCembly as a useful tool to teach spatial skills to visually impaired children. In all seven families, parents often transition between roles depending on their children's needs, favoring their autonomy in activities. When parents had the role of collaborators, children were more likely to reason aloud and work with the parent to find a solution to the task. The authors reported that parents played an important facilitating role both in spatial conceptualization, through spatial talk, and in perspective-taking, helping their children to correctly adopt the robot's frame of reference instead of their own. Finally, they underlined how parents also played a crucial role in facilitating learning and engagement by providing positive reinforcement to empower their children and keep them engrossed in the activity.

### 3.3 Parent involvement in RMI for pediatric patients with other medical conditions

Two studies investigated the engagement of parents in an RMI for pediatric patients with different medical conditions: only one of those was a controlled pilot study (#9), while the other one was an interpretive qualitative study (#6). The study's sample considers a range of ages comprised between 4 and 16 years; within this range, the most represented age average was 10 years. The population is female dominated.

The study by Okita et al. (#9) used Paro as a robotic tool designed to simulate animal-assisted intervention in hospital environments. It is equipped with five sensors: tactile, visual, auditory, temperature, and postural. Paro is thus able to recognize speech and caresses through its tactile and audio sensors, and it is able to detect reactive behaviors to reinforce mutual interaction. The other study by Butchart (#6) used the robot Nao, which we have already discussed in the previous paragraphs.

In the framework of therapeutic approaches for a reduction in perceived pain in the pediatric population, Butchart et al. (#6) aimed this study to analyze the experiences of parents and children during an RMI according to five themes: (a) affective influence, (b) independence, (c) preference for human interaction, (d) accessibility of therapy (only for parents), and (e) familiarity with technology. Parent–child pairs participated in up to three sessions with the NAO robot, lasting an average of 33 min each. Besides, Okita's study (#9) investigated whether this tool could reduce anxiety and pain in children and their parents and if the presence of a caregiver during intervention ameliorates the outcome. The 18 daughter–mother duos were randomly assigned to one of two conditions: with or without the presence of parents during the RMI session. They completed n. 1 session that lasted 30 min, during which they were invited to interact with Paro.

In both studies, the parent's perceptions and emotional aspects were assessed. Butchart et al. (#6) measured outcomes with semi-structured interviews; to examine the outcome, they used a process of inductive thematic review by coding the data and developing a concept map. Themes were generated from the initial coding by blinding different raters. For children, the semi-structured interview was implemented with the captioned picture method. Different questionnaires were used by Okita et al. (#9): the Wong-Baker FACES Pain Rating Scale was used for both parent and child to evaluate perceived pain. To determine both parent and child anxiety, the authors selected six items that overlap in two different questionnaires: the State-Trait Anxiety Inventory for Children (STAIC, Spielberger, 1973) and the adult version of the inventory and the State-Trait Anxiety Inventory (STAI, Spielberger et al., 1983).

In the study by Butchart et al. (2021), the results were characterized by a qualitative analysis of parent and child responses through interpretations of the data by different authors. No statistical data were described. Globally, the results showed that both parents and children affirmed the potential therapeutic value of the robot in supporting therapy programs, even if access to the treatment may be limited by distance from services and therapists' availability. Whereas, children perceived enjoyment and positive affective influence as the most important features of the NAO robot, the parent's affective experience resulted from their child's enhanced engagement and motivation, emphasizing the usefulness of the co-presence of parent and child during the rehabilitation sessions. Parents and children also considered the robot a useful tool that could improve the child's independence with the exercises through motivation, demonstration, coupled execution, and pacing while simultaneously alleviating parent-child tensions.

According to Okita's (2013) findings, the Paro robot would improve parental perspective through a shared experience: the direct interaction with Paro parents acknowledges their children's pain accurately. Moreover, this study highlights the importance of caregiver involvement: the 'with-parent' condition showed a significant decrease in patient-perceived pain, while no difference was observed in the 'alone' condition from pre- to post-intervention. Likewise, the 'with-parent' condition revealed a significant decrease in the patient's negative emotions by comparing the anxiety questionnaire scores.

### 3.4 Parent involvement in RMI for neurotypical children

Two publications reported parent presence in RMI with neurotypical children (ID #7, #8).

All the studies' samples consider a range of ages comprised between 1 and 9 years; within this range, the most represented age average was 4 years. The population was male dominated. Among those, only one study was a randomized controlled trial (#8), while the other one was an evaluation study (#7). The studies used a different robot to favor triadic interaction: the Nao robot (Tolksdorf et al., 2021) and Patricc (Gvirsman et al., 2020), a robotic platform designed for toddlers to entertain long-term interaction. This robot is characterized by a soft, puppet-like exterior that can be touched and felt to be more accessible for toddlers. The puppets can be “dressed,” and they can produce human-like gestures. The platform also includes a console for physical props toward which the robot can point and look to instigate joint attention with the toddler; finally, it provides an easy platform for the integration of content for non-programmers. In this study, Gvirsman et al. (ID #7) aimed to examine toddlers' and parents' interaction in an RMI with Patricc composed of educational activities. The authors evaluated the interactions of 18 parent-toddlers in three learning sessions lasting 8 min. “English as a Second Language” educational content was presented: in two sessions, the participants interacted with Patricc, and in one session, they interacted with a tablet and displayed a similar activity in a video with the same robot. Nine pairs interacted first with the tablet and subsequently with the robot, and vice versa for the remaining nine pairs; the pairs were assigned an order in a pseudo-randomized fashion. Similarly, Tolksdorf et al. (2021) intended to analyze caregiver involvement by children during a long-term interaction and whether this interaction changed during RMI. Following a between-subjects experimental design, the children were randomly assigned to a parallel learning situation with either the social robot or a human partner. All the participants and their caregivers attend four sessions, each lasting ~25–30 min, within 2 weeks. The learning situation was designed following the theoretical models of language pragmatics and multimodal communication and interaction (Rohlfing et al., 2016). A story containing novel target words embedded in the plot was told by either the robot or a human interlocutor (other than the parent). Then, the robot or the human asked the children to interact and play with targeted words. Both studies used video coding to measure interested behaviors. Specifically, in the study of Gvirsman et al. (2020), there are three gaze measures for each kind of participant: (a) children's gaze, directed to parents, the tablet, and the robot; and (b) parents' gaze toward the child, the tablet, and the robot. Conversely, Tolksdorf et al. (2021) measured non-verbal social referencing behavior using Vaish and Striano's (2004) paradigm: behaviors were coded during a predetermined time in which the robot and human interlocutors shared the story with new targeted words to learn. Moreover, to assess parental perceptions and provide qualitative assessments of their experience, Gvirsman et al. (2020) evaluated recorded and transcribed semi-structured interviews. The responses were coded and grouped into nine major and 10 minor themes. Following a within-subject design, Gvirsman et al. (2020) highlighted a significant increase in the parent's gaze at the toddler in the Patricc condition and significantly more mutual gazes at the tablet compared to the robot condition. The authors thus concluded that Patricc promoted significantly more triadic interaction between the toddler, parent, and robot than the tablet, which instead captivated most of the attention at the expense of the parent–toddler dyadic interaction. The qualitative analysis echoed these quantitative results, showing that 50% of parents perceived Patricc as a genuinely engaging and triadic parent–child activity. Tolksdorf et al. (2021) demonstrated that children socially refer to their caregiver in a novel educational setting with a social robot significantly more frequently than during an interaction with an unfamiliar human interlocutor, either for seeking reassurance or emotional support from their parent or to share their joy in having completed the task; finally, the authors stated that these implemented behaviors also persist across multiple sessions.

## 4 Discussion

The current literature considers parent-mediated intervention crucial for infant development, especially in preschool (Rayce et al., 2017). Thus, caregivers play an essential role in the child's development to ensure a healthy environment (Zeanah and Zeanah, 2009), and family involvement in a therapeutic setting provides continuity with the living environment and implies in the family itself an attitude of observation and modification (Toll et al., 2011). Over recent decades, many researchers have started to explore how new technology may implement innovative rehabilitation treatments. In the field of digital health, robot-assisted therapy has been used as a complementary therapy for children with various disabilities, in particular children with cerebral palsy (Ammann-Reiffer et al., 2017) and with ASD (Salhi et al., 2022). Numerous studies have already confirmed that the use of the robot is effective in pediatric rehabilitation (Gonzalez et al., 2021), particularly regarding improvement in social interaction, motivation, and aspects of social cognition such as emotion recognition and empathy (Diehl et al., 2012). A distinctive feature is the two-way interaction between the robot and the child, which improves attention skills while providing learning opportunities to children with special needs (Daniela, 2019). Moreover, the robot seems to increase motivation by providing engaging activities and supporting therapies that promote more complex social interactions to better mimic reality (Alabdulkareem et al., 2022).

Given the importance of parent-mediated interventions, we set out to perform a rigorous systematic review of parental involvement in RMI to verify the feasibility and usability of this powerful technology opportunity. To the best of our knowledge, this is the first literature review in this field. At the current state of the art, the articles reviewed seem to indicate the potential benefits of caregiver involvement in RMI. Besides, because of the small number of subjects in each experiment and the paucity of literature retrieved, this review cannot be considered a demonstration of the assumptions that have been exposed in our results.

The majority of the studies included focused on RMI in children with ASD, primarily examining the acceptability of RMI by parents and gathering parents' observations to measure intervention efficacy. Overall, the parents reported this intervention as helpful (ID #1, #4) and noted behavioral improvements not only in therapeutic settings but also in other environments (ID #3, #5). Specifically, these two studies reported an increase in social interactions, with positive changes in both verbal and non-verbal behaviors; this finding seems to support evidence from the literature that children with ASD demonstrate more social behaviors toward adults when an interaction partner is a robot rather than another adult (Chaminade et al., 2012; Damm et al., 2013; Kim et al., 2013). While social robots have been used as attractors or mediators in ASD, helping therapists establish easier connections with autistic individuals involving parental participation remains limited (Pennisi et al., 2016), the evidence supporting the efficacy of social robots for parental-involvement intervention in ASD is currently insufficient. More research is needed to substantiate the effectiveness of these interventions before they can be recommended for widespread clinical implementation.

In the field of pediatric medical conditions, the acceptability of RMI was confirmed in all the included studies. Moreover, two studies reported the role of parents as direct and indirect mediators of change (ID #2, #9). Specifically, involving the caregiver seems to enhance the parents' ability to empathize directly with their child and ameliorate parental modeling. This aspect may be relevant because children often cope with negative feelings by recalling how adults responded to similar situations (Muris et al., 1996; Gerull and Rapee, 2002). As the efficacy reported from only two studies is small overall, it could be an interesting factor to be taken into consideration in future research decision-making processes. Parental involvement in RMI could potentially play a different role in implementing efficacy and generalizing therapeutic improvement.

Overall, RMI certainly seems to play a facilitator role in improving caregiver educational skills. In fact, by manipulating the robot, the therapist can train the parent on specific behavioral techniques and simultaneously reinforce the child when he/she shows appropriate social initiations toward the parent (van den Berk-Smeekens et al., 2020; Butchart et al., 2021). The attention and engagement during robotic activities do not reduce throughout sessions in which the parent is present (Scasellati et al., 2018; van Otterdijk et al., 2020; Tolksdorf et al., 2021); furthermore, the parent's presence seems to increase children's involvement during RMI in cases of severe disorders such as severe autism with intellectual disability (Amirova et al., 2023). These data support, even more, the highly motivating feature of the tool and the desire for emotional sharing with the caregiver. Emotional sharing with the parent in the presence of a robotic tool increases social references (Tolksdorf et al., 2021), encouraging a systemic and integrated rehabilitation approach. Furthermore, sessions with the robot and the parent promote joint attention more significantly than other digital technologies, such as tablets (Gvirsman et al., 2020). This last aspect could depend on the interactive characteristics of the robot, which, unlike the tablet, do not decrease the frequency of dyadic interactions. Indeed, the results of our review support an increase in social reactivity toward the parent and a significant reduction in negative emotions (Okita, 2013). Furthermore, in pediatric diseases, RMI intervention could play an important role in improving the quality of daily life for the entire family (van Otterdijk et al., 2020). The feasibility and accessibility of this type of therapy do not only include outpatient settings; its use can also be arranged in home environments (Scasellati et al., 2018; Rocha et al., 2021), making it easily replicable. Besides, the studies reviewed had some limitations, such as small sample sizes and a greater presence of observational studies compared to RCTs. The main limitation concerns the unstructured involvement of caregivers within the RMI, limiting the evaluation of its effectiveness and not providing objective measures of change. In contrast, most studies have focused on parents' acceptance and adherence to RMI, emphasizing their opinion and degree of approval. Furthermore, generalizability was only evaluated in one study (Scasellati et al., 2018), where the broader influence of the robot-assisted intervention outside of intervention sessions was measured through a survey. These may be different crucial aspects on which to base future research, as our review highlights various potentials. However, these do not appear to be explored in depth with short-term interventions. Regarding parental presence, the use of a robotic tool serves to implement learning educational strategies and techniques.

Despite the limited results of this review, social robotic tools certainly have the potential to encourage a good degree of child participation. In RMI, parental involvement may help improve the child's social-communicative skills and generalize them in everyday life contexts. These could be several crucial aspects on which to base future research. Our review highlights several potentials, but these do not seem to have been explored in depth. Future studies should plan better-defined intervention programs to prove not only the feasibility but also the benefits and efficacy of specific aims with RCT design. Provision should be made for a better definition of the parental sample, with its temperamental and psychological features and specific measures inherent in parenting characteristics, to explore how parenting may change into this kind of intervention and which factors may be predictive of a good response. Moreover, another area of interest for future research could be the generalization and the long-term maintenance of expected effects, aspects investigated in only one study (Scasellati et al., 2018). Finally, it may be possible to adhere to robotic therapy in different settings, including the patient's own home, with a more comfortable and familiar environment for patients. Conversely, one of the intrinsic limitations of RMI may be the relatively higher costs of the tool and the need for adequate training to use/interact with the robot. This aspect, no less important, should also be investigated to create a realistic cost/benefit balance.

## 5 Limitations

This review has some limitations that must be taken into consideration when interpreting its results. Some of them are inherent to our research question: although the initial search yielded a large number of articles (1,106 results), there were few studies that investigated the specific involvement of caregivers in RMI. However, to our knowledge, this is the first and most comprehensive review in this field. The evidence available is often very heterogeneous, making it difficult to analyze parental contributions or involvement in detail. This gap presents an opportunity for further research on this topic. Regarding the limitations of the review process, we did not address the cost-effectiveness of the interventions because of the extreme variety of conditions considered in each study and because it was beyond the scope of the present study. A final significant limitation is related to the restricted number of databases used; however, we also hand-searched the references of the included articles or related reviews to expand the included literature. Many studies were difficult to classify, and most of them did not include a control group. Despite these challenges, the studies included are valuable for the type of innovative question we are addressing and can guide future research in this area.

## 6 Conclusions

This systematic review aimed to explore parent involvement in RMI to verify the benefits and limitations of this kind of intervention. The use of robotic systems in pediatric rehabilitation has its specific advantages. Notably, their similarity to playful activities makes them highly motivating for children and relevant to their technology-rich daily experiences. Moreover, the intensive and repetitive nature of robotic training activities stimulates neuronal plasticity, which is crucial for recovery. The review highlights that involving parents can significantly enhance the therapeutic process by addressing various goals and facilitating the generalization of learned behaviors. Integrating caregivers into robotic sessions not only supports children's learning but also creates an opportunity for shared educational experiences, strengthening the overall intervention.

Further research into robot-assisted interventions is essential to better understand their effectiveness, pinpoint specific areas of application, refine training protocols, and fully leverage the role of parents as integral resources in the therapeutic strategies that benefit children.

## Data availability statement

The raw data supporting the conclusions of this article will be made available by the authors, without undue reservation.

## Author contributions

AP: Writing – original draft, Methodology, Data curation, Conceptualization. CD: Writing – original draft, Conceptualization. MD: Writing – original draft, Formal analysis. CS: Writing – review & editing, Formal analysis, Data curation. FCo: Writing – original draft, Investigation. SL: Writing – review & editing. CI: Writing – review & editing. ET: Writing – review & editing. AQ: Writing – review & editing, Supervision, Project administration, Funding acquisition. FCu: Writing – review & editing, Validation, Supervision, Project administration.

## References

[B1] Akalin N. Loutfi A. (2021). Reinforcement learning approaches in social robotics. Sensors 21:1292. 10.3390/s21041292 33670257 PMC7918897

[B2] Alabdulkareem A. Alhakbani N. Al-Nafjan A. (2022). A systematic review of research on robot-assisted therapy for children with autism. Sensors 22:944. 10.3390/s22030944 35161697 PMC8840582

[B3] Amirova A. Rakhymbayeva N. Zhanatkyzy A. Telisheva Z. Sandygulova A. (2023). Effects of parental involvement in robot-assisted autism therapy. J. Autism Dev. Disorders 53, 438–455. 10.1007/s10803-022-05429-x 35088233 PMC9889445

[B4] Ammann-Reiffer C. Bastiaenen C. H. Meyer-Heim A. D. van Hedel H. J. (2017). Effectiveness of robot-assisted gait training in children with cerebral palsy: a bicenter, pragmatic, randomized, cross-over trial (PeLoGAIT). BMC Pediatr. 17:64. 10.1186/s12887-017-0815-y 28253887 PMC5333417

[B5] Baumrind D. (1985). Familial antecedents of adolescent drug use: a developmental perspective. NIDA Res. Monograph 56, 13–44. 10.1037/e468862004-001 3929097

[B6] Bean J. L. Eigsti I. M. (2012). Assessment of joint attention in school-age children and adolescents. Res. Autism Spectr. Disorders 6, 1304–1310. 10.1016/j.rasd.2012.04.003

[B7] Bearss K. Burrell T. L. Stewart L. Scahill L. (2015). Parent training in autism spectrum disorder: What's in a name?. Clin. Child Family Psychol. Rev. 18, 170–182. 10.1007/s10567-015-0179-5 25722072 PMC4516038

[B8] Benedetto L. (2005). Il Parent Training. Counseling e Formazione per Genitori. Carocci, 1–126.

[B9] Blizzard A. M. Barroso N. E. Ramos F. G. Graziano P. A. Bagner D. M. (2018). Behavioral parent training in infancy: What about the parent-infant relationship?. J. Clin. Child Adoles. Psychol. Off. Soc. Am. Psychol. Assoc. Div. 53, 47, S341–S353. 10.1080/15374416.2017.1310045 28414546 PMC5705575

[B10] Butchart J. Harrison R. Ritchie J. Martí F. McCarthy C. Knight S. . (2021). Child and parent perceptions of acceptability and therapeutic value of a socially assistive robot used during pediatric rehabilitation. Disab. Rehab., 43, 163–170. 10.1080/09638288.2019.1617357 31120794

[B11] Castillo J. C. Álvarez-Fernández D. Alonso-Martín F. Marques-Villarroya S. Salichs M. A. (2018). Social robotics in therapy of apraxia of speech. J. Healthcare Eng. 2018:7075290. 10.1155/2018/7075290 29713440 PMC5866898

[B12] Chaminade T. Da Fonseca D. Rosset D. Lutcher D. Cheng E. G. Deruelle C. (2012). “fMRI study of young adults with autism interacting with a humanoid robot,” in IEEE RO-MAN, 380–385.

[B13] Chronis A. M. Chacko A. Fabiano G. A. Wymbs B. T. Pelham W. E. Jr. (2004). Enhancements to the behavioral parent training paradigm for families of children with ADHD: review and future directions. Clin. Child Family Psychol. Rev. 7, 1–27. 10.1023/B:CCFP.0000020190.60808.a4 15119686

[B14] Coren E. Ramsbotham K. Gschwandtner M. (2018). Parent training interventions for parents with intellectual disability. The Cochr. Datab. Syst. Rev. 7:CD007987. 10.1002/14651858.CD007987.pub3 30004571 PMC6513025

[B15] Damm O. Malchus K. Jaecks P. Krach S. Paulus F. Naber M. . (2013). “Different gaze behavior in human-robot interaction in Asperger's syndrome: an eye-tracking study,” in IEEE RO-MAN, 368–369.

[B16] Daniela L. (2019). Smart Learning With Educational Robotics. Cham: Springer International Publishing.

[B17] Diehl J. J. Crowell C. R. Villano M. Wier K. Tang K. Riek L. D. . (2014). Clinical applications of robots in autism spectrum disorder diagnosis and treatment. Compr. Guide Autism 2, 411–422. 10.1007/978-1-4614-4788-7_14

[B18] Diehl J. J. Schmitt L. M. Villano M. Crowell C. R. (2012). The clinical use of robots for individuals with autism spectrum disorders: a critical review. Res. Autism Spectr. Disorders 6, 249–262. 10.1016/j.rasd.2011.05.006 22125579 PMC3223958

[B19] Duncan B. L. Miller S. D. Sparks J. A. Claud D. A. Reynolds L. R. Brown J. . (2003). The session rating scale: preliminary psychometric properties of a “working” alliance measure. J. Brief Ther. 3, 3–12.

[B20] English D. L. Gounden S. Dagher R. E. Chan S. F. Furlonger B. E. Anderson A. . (2017). Effects of video modeling with video feedback on vocational skills of adults with autism spectrum disorder. Dev. Neurorehab. 20, 511–524. 10.1080/17518423.2017.1282051 28632464

[B21] Fonagy P. (1998). Prevention, the appropriate target of infant psychotherapy. Infant Mental Health J. Off. Pub. World Assoc. Infant Mental Health 19, 124–150. 10.1002/(SICI)1097-0355(199822)19:2&lt;124::AID-IMHJ4&gt;3.0.CO;2-O

[B22] Forehand R. Jones D. J. Parent J. (2013). Behavioral parenting interventions for child disruptive behaviors and anxiety: what's different and what's the same. Clin. Psychol. Rev. 33, 133–145. 10.1016/j.cpr.2012.10.010 23178234 PMC3534895

[B23] Gardner F. Shaw D. S. Dishion T. J. Burton J. Supplee L. (2007). Randomized prevention trial for early conduct problems: effects on proactive parenting and links to toddler disruptive behavior. JFP Am. Psychol. Assoc. 21, 398–406. 10.1037/0893-3200.21.3.398 17874925

[B24] Gatta M. Balottin L. Mannarini S. Birocchi V. Del Col L. Battistella P. A. . (2016). Stress genitoriale e psicopatologia in età evolutiva. Uno studio caso-controllo [Parental Stress and psychopathological traits in children and adolescents. A controlled study]. Rivista di Psichiatr. 51, 251–259. 10.1708/2596.26726 27996985

[B25] Gerull F. C. Rapee R. M. (2002). Mother knows best: effects of maternal modelling on the acquisition of fear and avoidance behaviour in toddlers. Behav. Res. Ther. 40, 279–287. 10.1016/S0005-7967(01)00013-4 11863238

[B26] Gonzalez A. Garcia L. Kilby J. McNair P. (2021). Robotic devices for paediatric rehabilitation: a review of design features. Biomed. Eng. Online 20:89. 10.1186/s12938-021-00920-5 34488777 PMC8420060

[B27] Green J. Charman T. McConachie H. Aldred C. Slonims V. Howlin P. . (2010). Parent-mediated communication-focused treatment in children with autism (PACT): a randomised controlled trial. Lancet 375, 2152–2160. 10.1016/S0140-6736(10)60587-9 20494434 PMC2890859

[B28] Gvirsman O. Koren Y. Norman T. Gordon G. (2020). “Patricc: a platform for triadic interaction with changeable characters,” in Proceedings of the 2020 ACM/IEEE International Conference on Human- Robot Interaction (HRI '20), March 23–26, 2020, Cambridge, United Kingdom (New York: ACM), 9.

[B29] Hawkins J. D. Graham J. W. Maguin E. Abbott R. Hill K. G. Catalano R. F. . (1997). Exploring the effects of age of alcohol use initiation and psychosocial risk factors on subsequent alcohol misuse. J. Stud. Alcohol 58, 280–290. 10.15288/jsa.1997.58.280 9130220 PMC1894758

[B30] Kaminski J. W. Valle L. A. Filene J. H. Boyle C. L. (2008). A meta-analytic review of components associated with parent training program effectiveness. J. Abnorm. Child Psychol. 36, 567–589. 10.1007/s10802-007-9201-9 18205039

[B31] Kasari C. Gulsrud A. C. Wong C. Kwon S. Locke J. (2010). Randomized controlled caregiver mediated joint engagement intervention for toddlers with autism. J. Autism Dev. Disorders 40, 1045–1056. 10.1007/s10803-010-0955-5 20145986 PMC2922697

[B32] Kim E. Paul R. Shic F. Scassellati B. (2012). Bridging the research gap: Making HRI useful to individuals with autism. J. Hum. Robot Int. 22, 26–54. 10.5898/JHRI.1.1.Kim28396741

[B33] Kim E. S. Berkovits L. D. Bernier E. P. Leyzberg D. Shic F. Paul R. . (2013). Social robots as embedded reinforcers of social behavior in children with autism. J. Autism Dev. Disorders 43, 1038–1049. 10.1007/s10803-012-1645-2 23111617

[B34] Kroeger K. Sorensen R. (2010). A parent training model for toilet training children with autism. J. Int. Disab. Res. 54, 556–567. 10.1111/j.1365-2788.2010.01286.x 20576064

[B35] Laski K. E. Charlop M. H. Schreibman L. (1988). Training parents to use the natural language paradigm to increase their autistic children's speech. J. Appl. Behav. Anal. 21, 391–400. 10.1901/jaba.1988.21-391 3225256 PMC1286139

[B36] Lee J. Obinata G. (2013). “Developing therapeutic robot for children with autism: a study on exploring colour feedback,” in 2013 8th ACM/IEEE International Conference on Human-Robot Interaction (HRI). IEEE,173–174.

[B37] Muris P. Steerneman P. Merckelbach H. Meesters C. (1996). The role of parental fearfulness and modeling in children's fear. Behav. Res. Ther. 34, 265–268. 10.1016/0005-7967(95)00067-4 8881095

[B38] Murray L. (1990). “The impact of maternal depression on infant development,” in Dal Nascere al Divenire Nella Realta e Nella Fantasia, ed. L. de Cagno (Turin: Turin University).

[B39] Okita S. Y. (2013). Self-other's perspective taking: the use of therapeutic robot companions as social agents for reducing pain and anxiety in pediatric patients. Cyberpsychol. Behav. Soc. Netwo. 16, 436–441. 10.1089/cyber.2012.0513 23505968

[B40] Patterson G. R. DeBaryshe B. D. Ramsey E. (1989). A developmental perspective on antisocial behavior. The Am. Psychol. 44, 329–335. 10.1037/0003-066X.44.2.329 2653143

[B41] Pennisi P. Tonacci A. Tartarisco G. Billeci L. Ruta L. Gangemi S. . (2016). Autism and social robotics: a systematic review. Autism Res. Off. J. Int. Soc. Autism Res. 9, 165–183. 10.1002/aur.1527 26483270

[B42] Pires A. C. Rocha F. Barros Simão, A. J. Nicolau H. H. Guerreiro T. (2020). “Exploring accessible programming with educators and visually impaired children,” in Proceedings of the Interaction Design and Children Conference,148–160.

[B43] Postorino V. Sharp W. G. McCracken C. E. Bearss K. Burrell T. L. Evans A. N. . (2017). A systematic review and meta-analysis of parent training for disruptive behavior in children with autism spectrum disorder. Clin. Child Family Psychol. Rev. 20, 391–402. 10.1007/s10567-017-0237-2 28600643

[B44] Rakhymbayeva N. Amirova A. Sandygulova A. (2021). A long-term engagement with a social robot for autism therapy. Front. Robotics AI 8:669972. 10.3389/frobt.2021.669972 34222353 PMC8241906

[B45] Rayce S. B. Rasmussen I. S. Klest S. K. Patras J. Pontoppidan M. (2017). Effects of parenting interventions for at-risk parents with infants: a systematic review and meta-analyses. BMJ Open 7:e015707. 10.1136/bmjopen-2016-015707 29284713 PMC5770968

[B46] Reed H. E. McGrew S. G. Artibee K. Surdkya K. Goldman S. E. Frank K. . (2009). Parent-based sleep education workshops in autism. J. Child Neurol. 24, 936–945. 10.1177/0883073808331348 19491110 PMC3786206

[B47] Roberts J. M. Williams K. Smith K. Campbell L. (2016). Autism Spectrum Disorder: Evidence-Based/Evidence-Informed Good Practice for Supports Provided to Preschool Children, Their Families and Carers. London: Report prepared for the National Disability Insurance Agency (NDIA).

[B48] Roberts M. Y. Curtis P. R. Sone B. J. Hampton L. H. (2019). Association of parent training with child language development: a systematic review and meta-analysis. JAMA Pediatr. 173, 671–680. 10.1001/jamapediatrics.2019.1197 31107508 PMC6537769

[B49] Rocha F. Pires A. C. Neto I. Nicolau H. Guerreiro T. (2021). Accembly at home: accessible spatial programming for children with visual impairments and their families. Int. Design Children 2, 100–111. 10.1145/3459990.3460699

[B50] Rohlfing K. J. Wrede B. Vollmer A. L. Oudeyer P. Y. (2016). An alternative to mapping a word onto a concept in language acquisition: pragmatic frames. Front. Psychol. 7:470. 10.3389/fpsyg.2016.00470 27148105 PMC4835869

[B51] Rose S. D. (1974). Training parents in groups as behavior modifiers of their mentally retarded children. J. Behav. Ther. Exp. Psychiatr. 5, 135–140. 10.1016/0005-7916(74)90099-8

[B52] Rudovic O. Lee J. Mascarell-Maricic L. Schuller B. W. Picard R. W. (2017). Measuring engagement in robot-assisted autism therapy: a cross-cultural study. Front. Robotics AI 4:36. 10.3389/frobt.2017.00036

[B53] Salhi I. Qbadou M. Gouraguine S. Mansouri K. Lytridis C. Kaburlasos V. . (2022). Towards robot-assisted therapy for children with autism-the ontological knowledge models and reinforcement learning-based algorithms. Front. Robotics AI 9:713964. 10.3389/frobt.2022.713964 35462779 PMC9020227

[B54] Scasellati B. Boccanfuso L. Huang C. M. Mademtzi M. Qin M. Salomons N. . (2018). Improving social skills in children with ASD using a long-term, in-home social robot. Sci. Robotics3:eaat7544. 10.1126/scirobotics.aat7544 33141724 PMC10957097

[B55] Serketich W. J. Dumas J. E. (1996). The effectiveness of behavioral parent training to modify antisocial behavior in children: a meta-analysis. Behav. Ther. 27, 171–186. 10.1016/S0005-7894(96)80013-X28791693

[B56] Shaw D. S. Owens E. B. Giovannelli J. Winslow E. B. (2001). Infant and toddler pathways leading to early externalizing disorders. J. Am. Acad. Child Adoles. Psychiatr. 40, 36–43. 10.1097/00004583-200101000-00014 11195559

[B57] Spielberger C. D. (1973). Manual for the State-Trait Anxiety Inventory for Children. Palo Alto, CA: Consulting Psychologists Press. 10.1037/t06497-000

[B58] Spielberger C. D. Gorsuch R. L. Lushene R. Vagg P. R. Jacobs G. A. (1983). Manual for the State-Trait Anxiety Inventory. Palo Alto, CA: Consulting Psychologists Press.

[B59] Stein A. Gath D. H. Bucher J. Bond A. Day A. Cooper P. J. . (1991). The relationship between postnatal depression and mother child interaction. Br. J. Psychiatr. 158, 46–52. 10.1192/bjp.158.1.46 2015451

[B60] Steiner A. M. Koegel L. K. Koegel R. L. Ence W. A. (2012). Issues and theoretical constructs regarding parent education for autism spectrum disorders. J. Autism Dev. Disorders 42, 1218–1227. 10.1007/s10803-011-1194-0 21336525 PMC3810158

[B61] Sukhodolsky D. G. Smith S. D. McCauley S. A. Ibrahim K. Piasecka J. B. (2016). Behavioral interventions for anger, irritability, and aggression in children and adolescents. J. Child Adolescent Psychopharmacol. 26, 58–64. 10.1089/cap.2015.0120 26745682 PMC4808268

[B62] Thill S. Pop C. A. Belpaeme T. Ziemke T. Vanderborght B. (2012). Robot-assisted therapy for autism spectrum disorders with (partially) autonomous control: challenges and outlook. Paladyn 3, 209–217. 10.2478/s13230-013-0107-7

[B63] Tolksdorf N. F. Crawshaw C. E. Rohlfing K. J. (2021). Comparing the effects of a different social partner (social robot vs. human) on children's social referencing in interaction. Front. Educ. 5:569615. 10.3389/feduc.2020.569615

[B64] Toll S. W. Van der Ven S. H. Kroesbergen E. H. Van Luit J. E. (2011). Executive functions as predictors of math learning disabilities. J. Learn. Disab. 44, 521–532. 10.1177/0022219410387302 21177978

[B65] Tricco A. C. Lillie E. Zarin W. O'Brien K. K. Colquhoun H. Levac D. . (2018). PRISMA extension for scoping reviews (PRISMA-ScR): checklist and explanation. Annal. Int. Med. 169, 467–473. 10.7326/M18-0850 30178033

[B66] Vaish A. Striano T. (2004). Is visual reference necessary? Contributions of facial versus vocal cues in 12-month-olds' social referencing behavior. Dev. Sci. 7, 261–269. 10.1111/j.1467-7687.2004.00344.x 15595366

[B67] van den Berk-Smeekens I. van Dongen-Boomsma M. Korte D. Den Boer M. W. P. Oosterling J. C. Peters-Scheffer I. J. . (2020). Adherence and acceptability of a robot-assisted Pivotal Response Treatment protocol for children with autism spectrum disorder. Sci. Rep. 10:8110. 10.1038/s41598-020-65048-3 32415231 PMC7229010

[B68] van Otterdijk M. T. van den Berk-Smeekens M. W. Hendrix I. van Dongen-Boomsma J. Den Boer M. Barakova E. I. . (2020). The effects of long-term child–robot interaction on the attention and the engagement of children with autism. Robotics 9:79. 10.3390/robotics9040079

[B69] Wade C. Llewellyn G. Matthews J. (2008). Review of parent training interventions for parents with intellectual disability. J. Appl. Res. Int. Disab. 21, 351–366. 10.1111/j.1468-3148.2008.00449.x

[B70] Wainer A. L. Hepburn S. McMahon Griffith E. (2017). Remembering parents in parent-mediated early intervention: an approach to examining impact on parents and families. Autism Int. J. Res. Prac. 21, 5–17. 10.1177/1362361315622411 26951325

[B71] Wainer J. Ferrari E. Dautenhahn K. Robins B. (2010). The effectiveness of using a robotics class to foster collaboration among groups of children with autism in an exploratory study. Pers. Ubiquitous Comput. 14, 445–455. 10.1007/s00779-009-0266-z

[B72] Webster-Stratton C. H. Reid M. J. Beauchaine T. (2011). Combining parent and child training for young children with ADHD. J. Clin. Child Adoles. Psychol. Off. Soc. Child Adoles. Psychol. Am. Psychol. Assoc. Div. 53, 191–203. 10.1080/15374416.2011.546044 21391017 PMC3059849

[B73] Wetherby A. M. Guthrie W. Woods J. Schatschneider C. Holland R. D. Morgan L. . (2014). Parent-implemented social intervention for toddlers with autism: an RCT. Pediatrics 134, 1084–1093. 10.1542/peds.2014-0757 25367544 PMC4243066

[B74] Wong D. L. Baker C. M. (2012). Wong-Baker Faces Pain Rating Scale. London: Pain Management Nursing.

[B75] Wood L. J. Dautenhahn K. Lehmann H. Robins B. Rainer A. Syrdal D. S. . (2013). “Robot-mediated interviews: Do robots possess advantages over human interviewers when talking to children with special needs?,” in Social Robotics: 5th International Conference, ICSR 2013, Bristol, UK. Cham: Springer International Publishing, 54–63.

[B76] Yu J. Bai C. Roque R. (2020). “Considering parents in coding kit design: Understanding parents' perspectives and roles,” in Proceedings of the 2020 *CHI Conference on Human Factors in Computing Systems*, 1–14.

[B77] Zeanah C. H. Zeanah P. (2009). The Scope of Infant Mental Health Zeanah HJ, Handbook of Infant Mental Health. New York, NY: The Guilford Press.

[B78] Zwi M. Jones H. Thorgaard C. York A. Dennis J. A. (2011). Parent training interventions for attention deficit hyperactivity disorder (ADHD) in children aged 5 to 18 years. The Cochrane Datab. Syst. Rev. 2011:CD003018. 10.1002/14651858.CD003018.pub3 22161373 PMC6544776

